# Frailty and post-operative outcomes in older surgical patients: a systematic review

**DOI:** 10.1186/s12877-016-0329-8

**Published:** 2016-08-31

**Authors:** Hui-Shan Lin, J. N. Watts, N. M. Peel, R. E. Hubbard

**Affiliations:** Centre for Research in Geriatric Medicine, Princess Alexandra Hospital, The University of Queensland, Level 2, Building 33, Ipswich Road, Woolloongabba, QLD 4102 Australia

**Keywords:** Post-operative complications, Mortality, Geriatric, Oldest old, Frailty

## Abstract

**Background:**

As the population ages, increasing numbers of older adults are undergoing surgery. Frailty is prevalent in older adults and may be a better predictor of post-operative morbidity and mortality than chronological age. The aim of this review was to examine the impact of frailty on adverse outcomes in the ‘older old’ and ‘oldest old’ surgical patients.

**Methods:**

A systematic review was undertaken. Electronic databases from 2010 to 2015 were searched to identify articles which evaluated the relationship between frailty and post-operative outcomes in surgical populations with a mean age of 75 and older. Articles were excluded if they were in non-English languages or if frailty was measured using a single marker only. Demographic data, type of surgery performed, frailty measure and impact of frailty on adverse outcomes were extracted from the selected studies. Quality of the studies and risk of bias was assessed by the Epidemiological Appraisal Instrument.

**Results:**

Twenty-three studies were selected for the review and they were assessed as medium to high quality. The mean age ranged from 75 to 87 years, and included patients undergoing cardiac, oncological, general, vascular and hip fracture surgeries. There were 21 different instruments used to measure frailty. Regardless of how frailty was measured, the strongest evidence in terms of numbers of studies, consistency of results and study quality was for associations between frailty and increased mortality at 30 days, 90 days and one year follow-up, post-operative complications and length of stay. A small number of studies reported on discharge to institutional care, functional decline and lower quality of life after surgery, and also found a significant association with frailty.

**Conclusion:**

There was strong evidence that frailty in older-old and oldest-old surgical patients predicts post-operative mortality, complications, and prolonged length of stay. Frailty assessment may be a valuable tool in peri-operative assessment. It is possible that different frailty tools are best suited for different acuity and type of surgical patients. The association between frailty and return to pre-morbid function, discharge destination, and quality of life after surgery warrants further research.

## Background

As the population ages, the rate of surgical procedures in the older population is rising. In England, 2.5 million people over the age of 75 years underwent surgery between years 2014 and 2015, as opposed to just under 1.5 million between 2006 and 2007 [1, 2]. Nearly 30 % of these 2.5 million were over 85 years old [1]. Similarly, women aged 85 years and over now represent the largest proportion in emergency surgical admissions in Australia compared with all other age and sex groups [3].

It has long been recognised that advanced age can carry increased risk of mortality and morbidity after surgery. However, new knowledge is emerging that frailty, an age-related cumulative decline in multiple physiological systems, is a better predictor of mortality and morbidity than chronological age [4, 5]. Patients of the same age do not all have the same risk. The identification and assessment of frailty may facilitate identification of vulnerable surgical patients so that appropriate surgical and anaesthetic management can be implemented.

Experienced clinicians may feel that they can identify frailty by end-of-bed ‘gestalt’ assessments. However, ‘eyeballing’ is subjective and tends to be inconsistent between different observers [6]. Currently there is no standardised method of measuring frailty, with more than 20 different frailty instruments identified in a systematic review [7]. These different scales are based on the two main models which characterise how frailty develops and manifests. In the 'phenotype’ model described by Fried et al. [8], frailty manifests as decline in lean body mass, strength, endurance, balance, walking performance and low activity. Patients who have three or more of the five features of slowness, weakness, exhaustion, weight loss and low physical activity are deemed frail, while those who have none of the features are non-frail. Patients who display one or two of the five features are “pre-frail” [8].

The second model by Rockwood et al. is the Frailty Index (FI), or the cumulative deficit model, developed in the Canadian Study of Health and Aging (CSHA) [9]. This model conceptualises aging as the accumulation of deficits and views frailty as a multidimensional risk state quantified by the number of deficits rather than by the nature of the health problems. An FI can be based on comprehensive geriatric assessment and is calculated by counting the number of deficits present in an individual, divided by the total number of deficits measured [10]. The deficits encompass co-morbidities, physical and cognitive impairments, psychosocial risk factors and common geriatric syndromes [10]. The FI score ranges between 0 and 1, with higher scores indicating greater degree of frailty. FI represents a continuum; however, it can also be trichotomised to indicate low, intermediate and high level of frailty (FI ≤ 0.25, FI >0.25-0.4, FI >0.4) [11].

There has been a significant increase in literature over the last five years on the subject of frailty in surgical patients. A search for articles on Pubmed published between the years 2011 and 2015 using search terms ‘frailty’ AND ‘surgical outcome’ identified 173 titles, whereas the same search for publications between 2006 and 2010 yielded only 34 titles. The majority of the current literature investigating frailty and surgery has defined ‘geriatric’ as those above 60 or 65 years old. However, there has been a change in who is thought of as ‘old’. Basing studies on someone 65 years old may not provide insight into appropriate treatment for the ‘new’ geriatric patient [12]. Despite frailty being more prevalent with increasing age, and the large proportion of those over 75 years old undergoing surgery, frailty in the ‘old old’ and the ‘oldest old’ (aged 75–85 and over 85 years) surgical patients has been less comprehensively explored.

The aim of this systematic review, therefore, was to examine the association between frailty and adverse post-surgical outcomes in patients aged 75 years and over.

## Methods

### Search strategy

PUBMED, MEDLINE, EMBASE and Cochrane online databases were searched using search terms of ‘frail*’ AND ‘surg*’ in combination with (‘outcome’ OR ‘morbidity’ OR ‘complication’). An asterisk was used to indicate the term was truncated or had a variation in spelling. The search was conducted between October and December 2015 with filters applied to limit results to the English language, human research, and publications from year 2010 and onwards.

### Publication selection

The inclusion criteria for the search were: 1) the mean participant age was over 75 years; 2) the patient population had a surgical procedure; 3) frailty was assessed as a composite measure of more than one domain of health deficit, which accords with the current conceptualisation of frailty [13, 14] and was the main factor of interest in the study; and 4) the relationship between frailty and adverse outcomes was evaluated. Exclusion criteria were review articles, conference abstracts, and studies which measured frailty as a single item, such as a scan finding, a blood marker, or a physical performance test such as gait speed.

### Data extraction

Two reviewers (HL, JW) conducted the searches independently and compared results after assessing all identified abstracts for their compliance with the review criteria. Where agreement could not be reached a third independent reviewer (NP) was consulted. Reasons for exclusion were documented.

The following data were extracted from the eligible studies: sample size, mean age, country of origin of the study population, study design, type of surgery performed, frailty measure, and impact of frailty on adverse outcome.

### Assessment of study quality and risk of bias

Two reviewers (HL, JW) independently assessed the quality of the included studies using a modified version of the Epidemiological Appraisal Instrument (EAI), a valid and reliable tool for rating the quality of observational studies [15]. The EAI checklist addressed the following five domains of risk of bias: reporting, subject selection, measurement quality, data analysis, and generalisation of results. Each of the 23 questions in the EAI applicable to the selected studies was scored as yes (=2), partial (=1), no or unable to determine (=0) with the highest possible score being 46.

An a priori decision was made to divide the total possible score into quartiles. Quartile 1 (Q1) was 35–46 (the highest quality), quartile 2 (Q2) was 23–34, quartile 3 (Q3) was 12–23 and quartile 4 (Q4) was 0–11 (the lowest quality). Any disagreement regarding the assessment of the quality of a study was resolved by consulting a third reviewer (NP).

### Grading the overall strength of the evidence

The overall strength of the evidence was evaluated using principles outlined by the Agency for Healthcare Research and Quality [16]. The key elements of evaluation were quality (based on study design according to the hierarchy of evidence and study execution), quantity (based on the number of studies) and consistency.

## Results

**Fig. 1:**
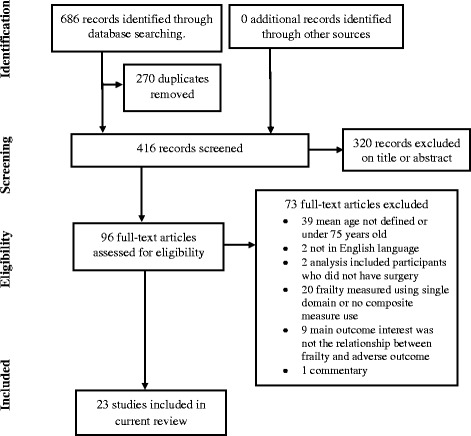
PRISMA flow diagram for study selection

The literature search identified 686 articles (187 from Pubmed, 169 from Medline, 300 from Embase and 28 from the Cochrane database). From these, 270 duplicate articles were removed. The titles, abstracts and the full texts of the articles were reviewed. Articles were selected based on inclusion and exclusion criteria. The references of selected articles were hand searched for further eligible articles. There were 23 articles included in the final analysis. The study selection process as well as the reasons for exclusion are shown in Fig. 1.

In the 23 articles selected for this review, there were 16 cohorts of patients with a mean or median age ranging from 75 to 87 years. Twenty studies were of prospective design with sample sizes ranging from 30 to 450 [17–36], and three were of retrospective design [37–39], one of which contained a large sample size of nearly 13,000 participants [37]. Publications came from different countries, including USA [17, 18, 35, 37–39], UK [30, 32, 34, 36], Europe [19–28, 31], and Asia [29, 33]. The proportion of females ranged from 31 % [34] to 83 % [35]. Five studies did not report the gender distribution of the cohorts [22, 23, 29, 32, 38]. A meta-analysis was not conducted due to a lack of homogeneity of frailty measures and the diversity of surgical procedures.

Nine studies measured frailty in cardiac surgery [17–24, 39], six in oncological surgery (predominantly focusing on colorectal cancer) [25–29, 37], three in general surgery [30, 31, 33], three in hip fracture surgery [35, 36, 38] and two in vascular surgery [32, 34]. Sixteen articles involved participants undergoing elective surgery [17–29, 33, 37, 39], five involved those undergoing acute surgery [30, 31, 35, 36, 38], while two included those undergoing both elective and acute surgery [32, 34]. Table 1, grouped by the type of surgery, describes the demographics, measurement of frailty and adverse outcome predicted by frailty for the selected studies.

**Table 1:** Study demographics grouped by type of surgery

Author	Sample size Country of origin Mean or median age % female Study design	Type of surgery	Frailty measure	Adverse outcome predicted by frailty	Association between frailty and adverse outcomes
Cardiac					
Afilalo, J et al. [17]^a^	152 USA, Canada Mean age 75.9 34 % female Prospective cohort study	Cardiac surgery (Elective)	Fried criteria (or Cardiovascular Health Study frailty scale) Modified CHS frailty scale *Fried + cognitive impairment + depressed mood* 4-item MSSA frailty scale *gait speed, handgrip strength, inactivity, cognitive impairment* Gait speed	Composite end point of post-operative mortality or major morbidity	Fried criteria, non-sig Modified CHS frailty scale, non-sig 4 item MSSA frailty scale, non-sig Gait speed, OR 2.63 (*p* < 0.05)
Green, P et al. [39]^a^	244 USA Median age, %female - frail 87.1,53 % - non-frail 85.4,45 % Post-hoc analysis of PARTNER trial	Transcatheter Aortic Valve Replacement (TAVR) (Elective)	Fried criteria condensed into 4 domains *gait speed, grip strength, serum albumin, Katz index of ADL* Frail ≥6/12	1) Adverse clinical events at 30 days 2) 1 year mortality 3) Poor outcome (composite mortality & QoL assessed by KCCQ-OS) a) 6 months b) 1 year	Adjusted for covariates 1) non-sig 2) OR 2.5 (*p* = 0.0002) 3) a) OR 2.21 (*p* = 0.03) b) OR 2.4 (*p* = 0.02)
Green, P. et al. [18]^b^	159 USA Mean age 86 50 % female Prospective cohort study	Transcatheter aortic valve replacement, (TAVR) (Elective)	Fried criteria condensed into 4 domains *gait speed, grip strength, serum albumin, Katz index of ADL* Frail >5/12	1) 1 year mortality 2) LOS 3) Procedural outcomes (any of major bleeding event, major vascular complications, stroke, acute kidney injury, 30 day mortality)	Adjusted for covariates 1) OR 3.5 (*p* = 0.006) 2) 9 vs 6 days (*p* = 0.004) 3) OR 2.2 (*p* = 0.04) for major bleeding but not other adverse outcomes
Kamga, M et al. [19]^b^	30 Belgium Mean age 86 47 % female Prospective cohort study	TAVI (Elective)	Score Hospitalier d'Evaluation du Risque de Perte d'Autonomie (SHERPA-risk of functional decline) score *MMSE, age, perceived poor health, fall in the last year, number of iADL independently performed before admission* Identification of Seniors at Risk (ISAR) score *>3 medications, self reported memory problems, sensory problems, hospital admission within the last 6 months, increased need for help at home*	1) 1 year mortality 2) Major cardiac and cerebral adverse events (MACCE)	Adjusted for covariates 1) SHERPA HR2.74 for every 1 point increase in score (*p* = 0.004) ISAR non-sig 2) SHERPA non-sig ISAR non-sig
Schoenenberger, A.W. et al. [20]^a^	119 Switzerland Mean age 83.4 55.5 % female Prospective cohort study	TAVI (Elective)	Mini Mental State Exam, Mini Nutritional Assessment, TUG, BADL, IADL, pre-clinical mobility disability Frail ≥3	1) Functional decline (BADL ↓ ≥1 point) 2) Functional decline or death among all participants at 6 months	Univariate 1) OR 3.31 (*p* = 0.02) 2) OR 4.46 (*p* = 0.001)
Stortecky, S. et al. [21]^a^	100 Switzerland Mean age 83.7 60 % female Prospective cohort study	TAVI (Elective)	Mini Mental State Exam, Mini Nutritional Assessment, TUG, BADL, IADL, pre-clinical mobility disability Frail ≥3	1) 30 day MACCE 2) 30 day mortality 3) 1 year MACCE 4) 1-year mortality	Univariate analysis 1) OR 4.78 (*p* = 0.05) 2) OR 8.33 (*p* = 0.03) 3) OR 4.89 (*p* = 0.003) 4) OR 3.68 (*p* = 0.02)
Sundermann S, et al. [22]^b^	400 Germany Mean age 80.3% female not reported Prospective cohort study	Cardiac surgery (Elective)	Comprehensive Assessment of Frailty *Fried minus unintentional weight loss, plus balance assessment, albumin, creatinine, brain natriuretic peptide, FEV1 and Clinical Frailty Scale* moderately frail = 11–25 points severely frail = 26–35 points	30 day mortality	Severely frail vs non frail 21.7 % vs 3.6 % AUC = 0.71 on logistic regression
Sundermann S, et al. [23]^b^	213 Germany Mean age 80.1 % female not reported Prospective cohort study	Cardiac surgery (Elective)	CAF FORECAST (Frailty predicts death One year after Elective Cardiac Surgery Tests)	1) 1 year mortality 2) Requirement for resuscitation 3) ICU stay 4) MACCE 1) 1 year mortality	Adjusted for EuroSCORE 1) OR 1.097 (*p* = 0.001) AUC 0.70 Frail vs non frail 2) 16 % vs 2 % (*p* < 0.05) 3) non-sig 4) non-sig 1) FORECAST AUC 0.76
Sundermann S, et al. [24]^b^	450 Germany Mean age 79 50 % female Prospective cohort study	Cardiac surgery (Elective)	CAF FORECAST *chair rise test, subjective weakness on questionnaire, stair climbing, Clinical Frail Scale and serum creatinine.*	1 year mortality	Adjusted for age CAF OR 1.091 (*p* < 0.001) FORECAST OR 1.265 (*p* < 0.001)
Oncologic					
Kristjansson S.R. et al. [25]^a^	178 Norway Mean age 79.63 57 % female Prospective cohort study	Colorectal cancer surgery (Elective)	Balducci Frailty Criteria from CGA *Cumulative Illness Rating Scale (CIRS), pADL, iADL, polypharmacy, MNA, MMSE, and GDS*	30 day post-operative complications (Clavian-Dindo grading)	Adjusted for covariates OR 3.13 (95 % CI 1.65–5.92)
Kristjansson S.R. et al. [26]^a^	176 Norway Mean age 80 57 % female Prospective longitudinal study	Cancer surgery (Elective)	Balducci Frailty Criteria from CGA Modified Fried criteria	30 day mortality	Adjusted for cancer stage and age Balducci OR 3.39 (*p* < 0.001) Modified Fried OR 2.67 (*p* = 0.029)
Neuman, H.B. et al. [37]^a^	12,979 USA Mean age 84.4 61.4 % female Retrospective analysis of Surveillance, Epidemiology and End Results(SEER)-Medicare database	Colectomy for stage I to III colon cancer (Elective)	11 item frailty measure defined by the John Hopkins Adjusted Clinical Group case-mix system *Difficulty walking, weight loss, frequent falls, malnutrition, impaired vision, decubitus ulcer, incontinence (plus 4 additional unnamed conditions)* Frail ≥1/11	1) 90 day survival 2) 1-year survival	Adjusted for covariates 1) OR 10.4 (*p* < 0.001) 2) OR 8.4 (*p* < 0.001)
Ommundsen, N. et al. [27]^a^	178 Norway Mean age 80 57 % female Prospective cohort study	Colorectal cancer surgery (Elective)	Balducci Frailty Criteria from CGA	5 year mortality	Multivariate adjusted for TNM stage and sex OR 3.6 (*p* < 0.001)
Ronning, B. et al. [28]^b^	84 Norway Median age 82 59 % female Prospective cohort study	Colorectal cancer surgery (Elective)	Balducci Frailty Criteria from CGA	Post-operative functional status 1) Barthel Index ↓ 2) NEADL ↓ 3) TUG ↑ 4) Grip strength ↓	Logistic regression (95 % CI) 1) non-sig 2) non-sig 3) non-sig 4) non-sig
Tan, K-Y et al. [29]^b^	83 Singapore and Japan Mean age 81.5 % female not reported Prospective cohort study	Colorectal cancer (Elective)	Fried criteria	Postop complications (Clavien-Dindo ≥ II)	Bivariate analysis OR 4.08 (*p* = 0.006)
General/abdominal					
Hewitt, J. et al. [30]^a^	325 UK Mean age 77.6 57 % female Prospective cohort study	General surgical patients (Acute) - only 31 % underwent surgery	Clinical Frailty Scale *7 frailty levels based on visual observation combined with an abbreviated review of medical records* Frail is ≥5	1) 30 day mortality 2) 90 day mortality 3) LOS 4) 30 day hospital readmission	Adjusted for age and polypharmacy, frail vs non frail 1) non-sig 2) non-sig 3) 19 vs 7 days (*p* = 0.02) 4) non-sig
Kenig, J et al. [31]^a^	184 Poland Mean age 76.9 53 % female Prospective cohort study	Abdominal surgery (Acute)	Vulnerable Elder Survey (VES) *age, self-rated health, limitation in physical function and functional disabilities* Triage Risk Screening Tool (TRST) *cognitive impairment, difficulty walking/transferring/recent falls, ≥5 medications, ED use in previous 30 days or hospitalization in previous 90 days, lives alone and/or no available caregiver, geriatric syndrome* G8 *7 items from the Mini Nutritional Assessment (MNA) questionnaire and age* Groningen Frailty Indicator (GFI) ADLs, sensory impairment, nutrition, polypharmacy, cognitive impairment, psychosocial wellbeing and subjective physical fitness Rockwood’s brief clinical instrument to classify frailty (4 frailty levels) Balducci Frailty Criteria	1) 30 day post-operative complications (Clavian-Dindo grading) 2) 30 day mortality	Adjusted for covariates 1) VES: OR 2.4 (*p* < 0.05) TRST: non-sig G8: OR 1.5 (*p* < 0.05) GFI: OR 1.5 (*p* < 0.05) Rockwood: non-sig Balducci: OR 1.7 (*p* < 0.05) 2) VES: OR 2.4 (*p* < 0.05) TRST: non-sig G8: OR 1.8 (*p* < 0.05) GFI: OR 1.4 (*p* < 0.05) Rockwood: non-sig Balducci: OR 1.4 (*p* < 0.05)
Kim, S et al. [33]^a^	275 Korea Mean age,% female - survivors 75.2, 46 % - deceased 77.6, 32 % Prospective cohort study	Intermediate or high risk general surgery (Elective)	Multidimensional Frailty Score (MFS) *Malignant disease, Charleston comorbidity Index, Albumin, ADLs, IADLs, dementia, risk of delirium, malnutrition, mid-arm circumference* Low risk ≤5 High risk >5	1) 1 year mortality 2) Discharge to residential care 3) Postoperative complications 4) LOS (median)	Adjusted for covariates, for every 1 point increase in MFS 1) OR 2.05 (*p* < 0.001) 2) OR 1.42 (*p* = 0.01) 3) non-sig 4) 14 vs 9 days for high vs low risk group (*p* < 0.001)
Vascular					
Ambler, G.K. et al. [32]^b^	410 UK Median age 77 % female not reported Prospective cohort study	Vascular surgery (Elective and Acute)	Addenbrooke’s Vascular Frailty Score (AVFS; 6 items, score 0–6) *Not independently mobile on admission, depression, polypharmacy on admission (>8 medications), anaemia, Waterlow score >13 on admission, emergency admission*	1) 1 year mortality 2) Readmission-free survival 3) Discharge to residential care 3) Prolonged LOS	Univariate; most vs least frail 1) 58 % vs 0 %, AUC 0.83 2) 0 % vs 68 % (*p* < 0⋅001), AUC 0.71 3) AUC 0.78 4) AUC 0.74
Partridge, J.S.L. et al. [34]^a^	125 UK Mean age 76.3 31 % female Prospective observational study	Vascular surgery (Elective and Acute)	Edmonton Frail Scale (EFS) *cognitive impairment, dependence in iADL, recent burden of illnesses, self-perceived health, depression, weight loss, medication issues, incontinence, inadequate social support and mobility difficulties.* Frail is >7/18	1) Composite measure post-operative complications 2) Composite measure adverse functional outcomes 3) LOS ≥12 days	Multivariate, adjusted for significant baseline associations and age 1) non-sig 2) non-sig 3) non-sig
Hip fracture					
Kistler, E et al. [35]^a^	35 USA Mean age 86 83 % female Prospective cohort study	Hip fracture surgery (Acute)	Modified Fried Criteria	1) Post-operative complications 2) Delirium 3) LOS 4) Time to surgery	Frail vs Non-frail 1) non-sig 2) non-sig 3) 7.3 vs 4.1 (*p* = 0.038) 4) non-sig
Krishnan, M et al. [36]^a^	178 UK Mean age 81 73.5 % female Prospective cohort study	Hip fracture surgery (Acute)	FI (51 items)	1) 30-day mortality 2) Inpatient mortality 3) LOS-failure to return home by 30 days	Frail vs Non-frail 1) 17.2 % vs 0 % (*p* < 0.001) 2) 28.1 % vs 0 % (*p* < 0.001) 3) AUC 0.82
Patel K.V. et al. [38]^a^	218 USA Mean age 81.2 % female not reported Retrospective chart review	Hip fracture (Acute)	Modified FI (19 items)	1 year mortality 2-year mortality	OR 4.97 (*p* < 0.001) OR 4.01 (*p* < 0.001)

^a^indicates quartile 1 in the quality assessment

^b^indicates quartile 2 in the quality assessment

*LOS* length of stay, *MACCE* major cardiac & cerebral adverse events, *non-sig* no statistically significant association, *AUC* area under the ROC curve for prediction of adverse outcomes

### Study quality and risk of bias

The EAI scores of the 23 studies ranged from 31 to 45, indicating they were in the upper two quartiles of study methodological quality. The EAI scores were in the in the second quartile for eight studies [18, 19, 22–24, 28, 29, 32] while the remainder 15 studies were in the first quartile [17, 20, 21, 25–27, 30, 31, 33–39]. There was a high level of agreement of quality assessment between the two independent reviewers. The most poorly reported items across all studies were: sample size calculation, adjustment for covariates and the report of losses to follow up. Study quality scores are incorporated into Table 2.

**Table 2:** Adverse outcome associated with frailty, grouped by the quality of studies

Outcome	Number of studies										
		1	2	3	4	5	6	7	8	9	10
Mortality											
1 year Mortality	Quality [ref]	**Q1** [21]	**Q1** [33]	**Q1** [39]	**Q1** [37]	**Q1** [38]	**Q2** [18]	**Q2** [19]	**Q2** [23]	**Q2** [24]	**Q2** [32]
*n* = 10	N sample	**100**	**275**	**244**	**12979**	**218**	**159**	**30**	**213**	**450**	**410**
2 Year Mortality	Quality [ref]	**Q1** [38]									
*n* = 1	N sample	**218**									
5 year Mortality	Quality [ref]	**Q1** [27]									
*n* = 1	N sample	**178**									
30 Day Mortality	Quality [ref]	**Q1** [21]	**Q1** [26]	**Q1** [31]	**Q1** [36]	**Q2** [22]	Q1 [30]				
*n* = 6	N sample	**100**	**176**	**184**	**178**	**400**	325				
90 Day Mortality	Quality [ref]	**Q1** [37]	Q1 [30]								
*n* = 2	N sample	**12979**	325								
Post-Operative Complications											
Non-routine recovery	Quality [ref]	**Q1** [25]	**Q1** [31]	**Q2** [18]	**Q2** [29]	Q1 [17]	Q1 [33]	Q1 [34]	Q1 [35]	Q1 [39]	
*n* = 10	N sample	**178**	**184**	**159**	**83**	152	275	125	35	244	
Need for resuscitation	Quality [ref]	**Q2** [23]									
*n =* 1	N sample	**213**									
Delirium	Quality [ref]	Q1 [35]									
*n* = 1	N sample	35									
MACCE	Quality [ref]	**Q1** [21]	Q2 [23]	Q2 [19]							
*n* = 3	N sample	**100**	213	30							
Discharge											
Length of stay	Quality [ref]	**Q1** [36]	**Q1** [35]	**Q1** [30]	**Q2** [32]	**Q2** [18]	Q1 [34]				
*n* = 6	N sample	**178**	**35**	**325**	**410**	**159**	125				
Discharge to Institution	Quality [ref]	**Q1** [33]	**Q2** [32]								
*n* = 3	N sample	**275**	**410**								
Functional Decline	Quality [ref]	Q1 [34]									
*n* = 1	N sample	125									
Post-Discharge											
Readmission rate: 1 year	Quality [ref]	**Q2** [32]	Q1 [30]								
*n* = 2	N sample	**410**	325								
Functional Decline	Quality [ref]	**Q1** [20]	Q2 [28]								
*n* = 2	N sample	**119** ***at 6 months***	84 *16*–*28 months*								
Quality of Life: 6 months, 1 year	Quality [ref]	**Q1** [39]									
*n* = 1	N sample	**244**									

Bold: studies which found statistically significant association

*Q1* quartile one quality assessment, *Q2* quartile two quality assessment, *MACCE* major cardiac & cerebral adverse events

### Frailty instruments

Of the 23 included studies, 21 different instruments were used to measure frailty. Variations of the Fried Criteria or instruments based on Comprehensive Geriatric Assessment (CGA), including the Frailty Index, were used in the majority of studies. Scales based on CGA are obtainable from patient interview as well as clinical notes without physical performance based measures, and were used in both acute and elective surgical cohorts. In contrast, the Fried frailty measure required physical performance-based tests, and was used exclusively in elective surgical cohorts. Four instruments, such as Multidimensional Frailty Score [33] and Comprehensive Assessment of Frailty [22–24], combined aspects of CGA with performance based tests (e.g. balance assessments, chair rise, stair climb) and medical investigations (e.g. blood test and respiratory function test). Details of measurement of frailty are presented in Table 1.

### Adverse outcomes predicted by frailty

Table 2 shows the adverse outcomes associated with frailty, grouped by the quality of the studies. Short, intermediate and long term mortality were assessed by 16 papers. Of ten studies evaluating the relationship between frailty and 12 month mortality, all found a significant relationship with frailty [18, 19, 21, 23, 24, 32, 33, 37–39]. Odds Ratios ranged between 1.1 and 4.97 for the frail patients compared with those who were non-frail [18, 21, 23, 24, 38, 39]. This association was found regardless of the instruments used to measure frailty and irrespective of the type of surgery performed.

In the two papers that assessed long term mortality, frailty was associated with increased two year mortality with an Odds Ratio of 4.01 [38] and increased five year mortality with an Odds Ratio of 3.6 [27]. The association between frailty and 90 day mortality was evaluated in two studies [30, 37]. One found a significant association with an Odds Ratio of 10.4 [37] while the other did not find a significant association [30]. Thirty day mortality was evaluated in six studies [21, 22, 26, 30, 31, 36]; all but one [30] found a significant association, with Odds Ratios ranging between 1.4 and 8.33 [21, 26, 31]. This latter study included only a small proportion (31 %, *n* = 105) of patients who underwent surgery [30].

Post-operative complications, as graded by the Clavian-Dindo severity classification [40] or pre-defined by the authors, were evaluated in nine papers [17, 18, 25, 29, 31, 33–35, 39]. Frailty was associated with increased post-operative complications in four studies with Odds Ratios ranging from 1.5 to 4.8 [18, 25, 29, 31]. The remaining five studies reported no significant association [17, 33–35, 39]. The definitions used for post-operative complications in these 10 studies were heterogeneous. Conditions pre-specified in the studies which counted as a post-operative complication included cardiac complications (namely myocardial infarction, heart failure, arrhythmia), pulmonary embolism, pneumonia, wound infection, major bleeding, renal failure, delirium, unplanned return to theatre and unplanned intensive care unit admission.

Specific items of post-operative complications were also examined by several studies. An association between frailty and major cardiac and cerebral adverse events (MACCE) was reported by one of the three studies evaluating this outcome [19, 21, 23]. One study explored the association between frailty and delirium and did not find a significant association [35]. Of two studies evaluating frailty and readmission rate, one study found a significant association [32] while the other did not [30]. One study showed a significant association between frailty and the need for resuscitation [23].

Of the six studies that included prolonged length of stay as an outcome, an association with frailty was found in five [18, 30, 32, 35, 36]. Three studies evaluated functional decline as an outcome, of which only one found a significant association [20]. Discharge to a residential care facility was found to be associated with frailty by both studies in which this outcome was evaluated [32, 33]. Quality of life was evaluated in one study and frailty was associated with the composite poor outcome of mortality or poorer quality of life [39].

Based on quality, quantity and consistency of the included studies, there is evidence for an association between frailty and adverse postoperative outcomes. Although cohort studies are lower on the hierarchy of evidence than randomised controlled trials, it is acknowledged that the cohort study design is entirely appropriate for investigating this particular research question. The literature search identified 23 studies that met the inclusion criteria and 15 of those were in the upper quartile of quality assessment, indicating the majority were methodologically sound. The consistency was evidenced by the finding that 20 of the included studies found evidence of an association between frailty and at least one adverse outcome.

## Discussion

The reviewed studies consistently found that in patients aged over 75 years, frailty was associated with increased mortality, post-operative complications, prolonged length of stay and discharge to residential care facility. The strongest evidence of association was between frailty and 30 day mortality. The association was consistent across different frailty instruments and regardless of the type of surgery performed.

Our findings are congruent with other reviews of frailty in surgical patients. Beggs et al. found eight out of 19 articles demonstrating frailty to be significantly associated with mortality and post-operative complications [41]. Other systematic reviews have concentrated on specific surgical subspecialties, namely oncologic surgery [42], cardiac surgery [43] and thoracic surgery [44]. They also found frailty to impact negatively on post-operative outcomes. Two other reviews written on cardiac surgery also identified frailty as a risk factor that provided important prognostic information in older adults needing surgical or transcatheter aortic valve replacement [45] and found that frailty increased the predictive power of conventional risk scores [46].

The strength of this review is that it is inclusive of all types of surgery, both elective and acute, and focuses on those over 75 years old. This review provided insight into how frailty is measured and how it correlates with adverse outcomes in the ‘old-old’ and the ‘oldest old’ surgical population. Our search was limited to English publications, so may have excluded relevant publications in other languages. Another limitation was that studies using single markers to determine frailty, such as measurement of muscle mass or gait speed, were excluded based on the consensus view of frailty being a multidimensional state of increased vulnerability. Finally, due to the differences in frailty instruments used and heterogeneity of the surgical patient population, meta-analysis could not be conducted, and the magnitude of the adverse impact of frailty on outcome could not be estimated.

There is evidence that frailty is associated with increased mortality and morbidity in the older surgical patients. As patients over 75 years old are presenting more commonly for surgery, frailty assessment may have considerable value as a tool for peri-operative assessment. However, for the value of frailty assessment to be realised, it must not only predict outcomes but also be easily incorporated into routine assessment or created from existing information, without placing further resource burden on clinical staff and the patient. Once established, such a tool may offer a valuable addition to the risk assessment of older persons undergoing surgery, alongside the standard surgical and anaesthetic assessment tools. With the increasing focus on patient centred care, the ongoing development of frailty assessment has the potential to improve how well patients can be informed by their surgeons and anaesthetists prior to their procedures, thus enhancing informed consent. The clinical utility, time taken to make frailty assessments and the ease of use of most of the tools in the 23 included studies were not reported, which would be useful in assisting clinicians to decide which tool to adopt into clinical practice.

This review found several important gaps in the current literature. Frailty in acute surgical patients is under-studied. Only 7 out of 23 studies assessed acute surgical patients and all of them used scales based on comprehensive geriatric assessment to measure frailty. Reliance on performance based tests may be impractical in the acute surgical patients. More research into how frailty impacts on surgical patients in the acute setting and how best to measure frailty in acute surgical patients is needed. An instrument which is robust and valid for measuring frailty in elective patients in a surgical pre-admission clinic may not be applicable to the acute patients. Despite the need to find a unified tool for measuring frailty, it is possible that different frailty tools are best suited for different acuity and type of surgical patients. Furthermore, these instruments need to be time-efficient and suitable for application at the bedside by staff who are not geriatricians.

Mortality and post-operative complications are the most commonly studied and reported outcomes in the 23 articles reviewed. Quality of life post-surgery was assessed in only one out of the 23 studies; similarly, functional decline and discharge to a care facility were only evaluated in three and two studies respectively. The association between frailty and functional outcome, discharge destination, and quality of life after surgery warrants further research. Factors and outcomes important to the individual elderly patient undergoing surgery must also be considered when performing pre-operative assessment, such as the consideration of premorbid status and return to the premorbid level of function.

## Conclusion

Frailty is consistently found is to be associated with adverse outcomes after surgery. In the 23 articles reviewed, the strongest evidence lies in the association with increased 30 day, 90 day and 1 year mortality, post-operative complications and length of stay. This highlights the importance of detecting frailty in peri-operative assessment. The possibility that different frailty tools may be best suited for different acuity and type of surgical patients is worth exploring. The association between frailty and return to pre-morbid function, discharge destination, and quality of life after surgery warrants further research.

## Abbreviations

FI: Frailty index
CSHA: Canadian Study of Health and Aging
EAI: Epidemiological appraisal instrument
CGA: Comprehensive geriatric assessment
MACCE: Major cardiac and cerebral adverse events
